# Absolute Quantification of Major Photosynthetic Protein Complexes in *Chlamydomonas reinhardtii* Using Quantification Concatamers (QconCATs)

**DOI:** 10.3389/fpls.2018.01265

**Published:** 2018-08-30

**Authors:** Alexander Hammel, David Zimmer, Frederik Sommer, Timo Mühlhaus, Michael Schroda

**Affiliations:** Molekulare Biotechnologie und Systembiologie, Technische Universität Kaiserslautern, Kaiserslautern, Germany

**Keywords:** mass spectrometry, proteotypic peptide, QconCATs, photosynthesis, light reactions, pyrenoid, Rubisco, *Chlamydomonas reinhardtii*

## Abstract

For modeling approaches in systems biology, knowledge of the absolute abundances of cellular proteins is essential. One way to gain this knowledge is the use of quantification concatamers (QconCATs), which are synthetic proteins consisting of proteotypic peptides derived from the target proteins to be quantified. The QconCAT protein is labeled with a heavy isotope upon expression in *E. coli* and known amounts of the purified protein are spiked into a whole cell protein extract. Upon tryptic digestion, labeled and unlabeled peptides are released from the QconCAT protein and the native proteins, respectively, and both are quantified by LC-MS/MS. The labeled Q-peptides then serve as standards for determining the absolute quantity of the native peptides/proteins. Here, we have applied the QconCAT approach to *Chlamydomonas reinhardtii* for the absolute quantification of the major proteins and protein complexes driving photosynthetic light reactions in the thylakoid membranes and carbon fixation in the pyrenoid. We found that with 25.2 attomol/cell the Rubisco large subunit makes up 6.6% of all proteins in a *Chlamydomonas* cell and with this exceeds the amount of the small subunit by a factor of 1.56. EPYC1, which links Rubisco to form the pyrenoid, is eight times less abundant than RBCS, and Rubisco activase is 32-times less abundant than RBCS. With 5.2 attomol/cell, photosystem II is the most abundant complex involved in the photosynthetic light reactions, followed by plastocyanin, photosystem I and the cytochrome b_6_/*f* complex, which range between 2.9 and 3.5 attomol/cell. The least abundant complex is the ATP synthase with 2 attomol/cell. While applying the QconCAT approach, we have been able to identify many potential pitfalls associated with this technique. We analyze and discuss these pitfalls in detail and provide an optimized workflow for future applications of this technique.

## Introduction

*Chlamydomonas reinhardtii* has been used since many decades as a model system to study various aspects of cell biology (Harris, 2008). Recent advancements, like the development of robust protocols for genome editing (Ferenczi et al., 2017; Greiner et al., 2017) or the establishment of an indexed mutant library comprising about 60,000 mutants (Li et al., 2016), will further boost *Chlamydomonas* as a plant model system. As a unicellular green alga, *Chlamydomonas* is particularly suited for plant systems biology approaches (Hemme et al., 2014). Important especially for mathematical modeling in systems biology is the knowledge of the absolute concentrations of biomolecules in a cell (Pratt et al., 2006). For proteins, this information is difficult to obtain and before the introduction of mass spectrometry was done for example by photospectroscopy on proteins harboring light-absorbing cofactors, by radioligand assays, or by immunoassays requiring protein standards (Merchant et al., 1991; Murakami et al., 1997; Willmund et al., 2008).

Mass spectrometry-based shotgun or discovery proteomics aims at identifying a large number of cellular proteins and allows to quantify changes in the abundance of a subset of these proteins e.g., upon changing environmental conditions (Gillet et al., 2016). However, because standards are missing, this approach does not allow determining the absolute abundance of a protein within a cell. A common way to achieve this is to spike a known amount of synthetic peptides that mimic peptides produced by the proteolytic cleavage of target analyte proteins, into a whole cell protein extract. Either the synthetic peptides or the proteins in the extract are labeled with stable isotopes, thus leading to light and heavy peptide pairs after proteolytic cleavage. After ionization, these pairs can be separated and quantified by mass spectrometry, with the synthetic peptide serving as calibrator (Barnidge et al., 2003; Gerber et al., 2003). This approach has already been applied to *Chlamydomonas*: in one study, PSI-LHCI complexes were isolated from *Chlamydomonas* cells that had been metabolically labeled by feeding an arginine-auxotrophic strain with ^13^C-arginine. Known amounts of unlabeled proteotypic peptides from PSI and LHCI were then added to purified ^13^C-labeled PSI-LHCI complexes and protein stoichiometries in the complex determined (Stauber et al., 2009). In two more studies, isotope-labeled peptides were spiked into extracts from unlabeled *Chlamydomonas* cells to determine absolute abundances of proteins involved in a variety of cellular processes (Wienkoop et al., 2010; Recuenco-Munoz et al., 2014). The advantage of these approaches is that they allow the absolute quantification of several target proteins in a single MS run. The disadvantages are the cost of the synthetic peptides, especially if they need to be synthesized with stable isotopes for a large number of target proteins, and the difficulty to quantify these peptides accurately, because of their tendency to irreversibly adhere to vessel walls (Brownridge et al., 2011).

These problems may be circumvented by using so-called quantification concatamers (QconCATs) (Beynon et al., 2005; Pratt et al., 2006). QconCATs consist of concatenated proteotypic peptides, an affinity tag allowing purification under denaturing conditions (usually a hexa-histidine tag) and, optionally, amino acids like cysteine or tryptophane for easy quantification. A QconCAT protein is expressed in *E. coli* from an *in silico* designed, codon-optimized synthetic gene cloned into an expression vector. A defined amount of the QconCAT protein is then added to the complex sample and, upon tryptic digestion, the proteotypic peptides from the QconCAT protein are released together with the corresponding peptides from the parent proteins. All QconCAT peptides are present in a strict 1:1 ratio at the concentration determined for the entire protein. The QconCAT protein can be heavy labeled in *E. coli*, or the unlabeled protein added to labeled proteins from the target organism. A QconCAT protein has been used to quantify changes in the abundance of vacuolar transporters in tonoplast-enriched fractions from leaves of *Arabidopsis thaliana* plants exposed to salinity and drought (Pertl-Obermeyer et al., 2016). However, we are not aware of any reports on the use of QconCAT proteins for the absolute quantification of proteins in *Chlamydomonas*.

Here, we report on the application of the QconCAT approach to *Chlamydomonas reinhardtii* for the absolute quantification of major proteins and protein complexes in the thylakoid membranes and in the pyrenoid that carry out photosynthetic light reactions and carbon fixation, respectively. We analyze problems encountered with the QconCAT approach and provide recommendations for labs interested in applying this technique to *Chlamydomonas* or other organisms.

## Materials and Methods

### Growth of *Chlamydomonas* Cells

Cells of *Chlamydomonas reinhardtii* strain CC-1883 were grown in TAP medium (Kropat et al., 2011) at a light intensity of 30 μmol photons m^-2^ s^-1^ to mid log phase. Cells were harvested by centrifugation for 2 min at 3,000 × g and 4°C, resuspended in 25 mM NH_4_HCO_3_, aliquoted at a final cell density of about 5.9 × 10^8^ cells ml^-1^, snap-frozen in liquid nitrogen, and stored at -20°C. The total protein concentration was measured according to Lowry et al. (1951).

### QconCAT Protein Expression and Purification

The coding sequence for the photosynthesis QconCAT protein (PS-Qprot) was codon-optimized for *E. coli*, synthesized by Biocat (Heidelberg) harboring BamHI/HindIII restriction sites, cloned into the pET-21b expression vector (Novagen), and transformed into *E. coli* ER2566 cells (New England Biolabs). For ^15^N-labeling of the PS-Qprot, M9 minimal medium was prepared with 9.2 mM ^15^NH_4_Cl (98%, Cambridge Isotope Laboratories) and 100 μg ml^-1^ Ampicillin. Transformed ER2566 cells were grown overnight at 37°C in ^15^N-M9 medium (Scott et al., 2016), diluted 1:50 into 300 ml ^15^N-M9 medium and grown to an OD of ∼0.8 when protein expression was induced by adding IPTG to a final concentration of 0.5 mM. After another 5 h at 37°C, cells were harvested by centrifugation for 10 min at 6,000 × g and 4°C and the cell pellet was stored at -20°C. Cells were lysed by sonication in 6 M guanidine-HCl, 20 mM Tris-HCl pH 8, 0.25 M NaCl, 5 mM imidazole and cleared by a 20-min centrifugation at 13,000 × g and 20°C. The supernatant was applied to a Co-NTA column (G-Biosciences) followed by three washes with Urea Buffer (8 M urea, 20 mM Tris-HCl pH 8, 0.25 M NaCl) containing 5 mM, 25 mM, and 100 mM imidazole, respectively. The PS-Qprot was then eluted with Urea Buffer containing 500 mM imidazole. Next, the eluted protein was electrophoresed on a preparative 12% SDS-polyacrylamide gel and the gel was negatively stained with 0.3 M CuCl_2_. The protein band was excised and, after three washes with 0.25 mM EDTA, 0.25 mM Tris-HCl pH 9 for destaining, the PS-Qprot was electroeluted by placing the gel slice in Laemmli Buffer (24.8 mM Tris, 134.2 mM glycin, 0.1% SDS and 1 mM EDTA) into a dialysis bag with 3.5 kDa MWCO (Spectrum Inc.) and applying 100 V for 1 h. The eluted protein was concentrated and dialyzed into phosphate-buffered saline. The protein concentration was determined spectroscopically at 280 nm on a NanoDrop spectrophotometer based on the Lambert-Beer’s law assuming a molecular weight of the PS-Qprot of 39,945.63 and an extinction coefficient of 86,860 M^-1^ cm^-1^. The latter were determined with the ExPASy ProtParam tool^1^. The protein concentration was adjusted to 1 μg/μl and the protein was stored at -20°C.

### In Solution Tryptic Digest and LC-MS/MS Analysis

Fifty micrograms of total *C. reinhardtii* protein (corresponding to 3.87 × 10^6^ cells) were mixed with 0.25, 0.5, 2.5, and 5 μg PS-Qprot, respectively, and precipitated overnight at -20°C after adding ice-cold acetone to a final volume of 80%. 1 μg of the PS-Qprot were also precipitated with acetone without *C. reinhardtii* protein to obtain a total ion count of the Q-peptides alone. Precipitated proteins were pelleted by centrifugation for 20 min at 25,000 × *g* and 4°C. After washing with 80% acetone, the pelleted proteins were air-dried and resuspended in 8 M urea, 25 mM NH_4_HCO_3_. The samples were then supplied with DTT at a final concentration of 12.5 mM, incubated for 30 min at 25°C and, to carboxymethylate reduced thiols, incubated for another 20 min in the dark in the presence of iodoacetamide at a final concentration of 25 mM. After diluting samples with 25 mM NH_4_HCO_3_ to a final urea concentration of 4 M, Lys-C was added at a ratio of 1:100 (w/w, Lys-C to protein) and digestion allowed to take place for at least 2 h at 37°C. Samples were further diluted with 25 mM NH_4_HCO_3_ to a final urea concentration of 1 M and supplemented with acetonitrile to a final concentration of 5%. Trypsin was then added at a ratio of 1:100 (w/w, trypsin to protein) and proteins allowed to digest overnight at 37°C. To complete digestion, more trypsin was added to yield a ratio of 1:50 and the samples incubated for another 3 h at 37°C. Digestion was terminated by adding formic acid at a final concentration of 2%. Tryptic peptides were desalted on home-made C18-STAGE tips (Empore), eluted with a solution of 80% acetonitrile/2% formic acid, dried to completion in a speed vac and stored at -20°C. Peptides were resuspended in a solution of 2% acetonitrile, 2% formic acid just before the LC-MS/MS run. The LC-MS/MS system (Eksigent nanoLC 425 coupled to a TripleTOF 6600, ABSciex) was operated in μ-flow mode using a 25 μ-emitter needle in the ESI source. Peptides were separated by reversed phase (Triart C18, 5 μm particles, 0.5 mm × 5 mm as trapping column and Triart C18, 3 μm particles, 300 μm × 150 mm as analytical column, YMC) using a flow rate of 4 μl/min and gradients from 2 to 35% HPLC buffer B (buffer A 2% acetonitrile, 0.1% formic acid; buffer B 90% acetonitrile, 0.1% formic acid). The efficiency of ^15^N incorporation in the labeled peptides was estimated according to Schaff et al. (2008). The intensities for the monoisotopic, fully ^15^N labeled peak (M_i_) and the preceding, first unlabeled peak (M_i-1_), containing one ^14^N, were extracted using PeakView v2.2 software (ABSciex) and used for calculating the labeling efficiency. BioFsharp^2^ was used for the extraction of ion chromatograms and for the quantification of peak areas of heavy Q-peptides and light native peptides. Assuming similar ionization properties for non-oxidized and oxidized methionine-containing peptides, the percentage of methionine oxidation was determined by extracting XICs for non-oxidized and oxidized (+16 amu) ^14^N peptides (% oxidation of native peptides) and non-oxidized and oxidized (+16 amu) ^15^N peptides (% oxidation of Q-peptides) (**Supplementary Dataset 1**). The raw data of our study have been uploaded to PeptideAtlas with the identifier PASS01212.

## Results

### The PS-Qprot Targets Major Soluble and Membrane-Intrinsic Photosynthesis Proteins in *Chlamydomonas*

**FIGURE 1:**
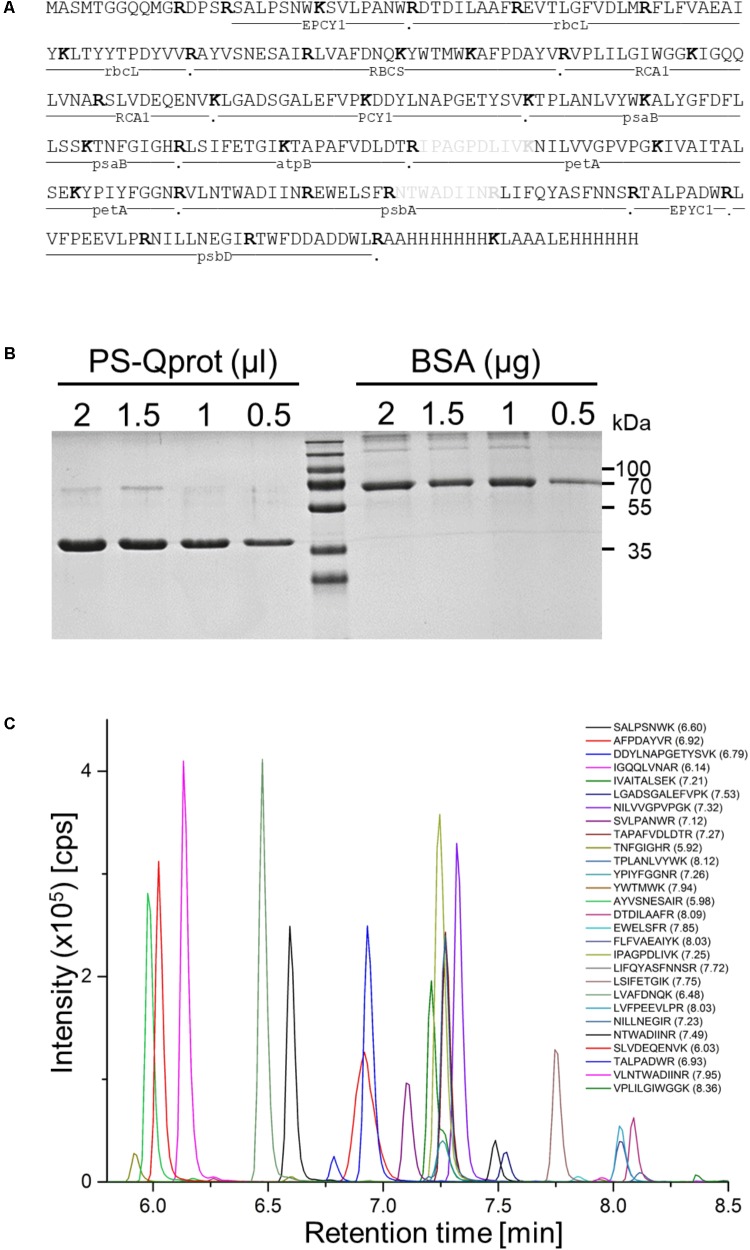
Properties of the PS-Qprot and its Q-peptides. **(A)** Sequence of the PS-Qprot and protein source of selected Q-peptides. Peptides in gray were erroneously included and have no native partner peptide. **(B)** The purified, ^15^N-labeled PS-Qprot was quantified on a NanoDrop spectrophotometer and the concentration adjusted to 1 μg/μl. The indicated volumes of the PS-Qprot were then separated next to a BSA standard on a 12%-SDS polyacrylamide gel and stained with Coomassie blue. **(C)** Extracted ion chromatograms (XICs) of the proteotypic ^15^N labeled Q-peptides derived from the PS-Qprot. The purified protein was tryptically digested and run on a short 6-min HPLC gradient. XICs of the resolved peptides were extracted using the PeakView software (ABSciex). The apex of the elution time is given in parentheses behind the peptide sequence. Note that due to the very short run only 28 of the 32 peptides were detected within the retention time window.

To test the applicability of QconCAT proteins for the absolute quantification of membrane-intrinsic and soluble proteins in *Chlamydomonas reinhardtii*, we chose to focus on the major protein complexes driving the photosynthetic light reactions in the thylakoid membranes, as well as the major proteins responsible for CO_2_ fixation in the pyrenoid. The former are photosystems (PS) I and II, the cytochrome b_6_/*f* complex, plastocyanin, and the ATP synthase (Eberhard et al., 2008). The latter are Rubisco, the Essential Pyrenoid Component 1 (EPYC1, also known as LCI5) that links Rubisco to form the pyrenoid, and Rubisco activase (Mackinder et al., 2016). While plastocyanin, EPYC1 and Rubisco activase are monomers or homooligomers, the other ones represent core subunits of heterooligomeric complexes, i.e., psbA and psbD of PSII, psaB of PSI, petA of the cytochrome b_6_/*f* complex, atpB of the ATP synthase, and rbcL and RBCS of Rubisco. For each of these 10 proteins analyzed we selected two to four different proteotypic tryptic (Q-)peptides in the mass range of 700–3,000 Da that in earlier studies have been detected by LC-MS/MS with good ion intensities and normal retention times. Selected RBCS Q-peptides did not distinguish between RBCS1 and RBCS2. Hence, these peptides were likely detectable by LC-MS, but there may have been ones with better ionization propensities. This photosynthesis QconCAT protein (PS-Qprot) also contained two sacrificial tryptic peptides at the N-terminus with the methionine initiator amino acid, and two more at the C-terminus, each containing a hexa-histidine tag (**Figure 1A**).

### The Measured Ratios of Q-Peptides to Native Peptides Correlate Well With the Ratios at Which the PS-Qprot Was Added to *Chlamydomonas* Cell Extract

The 39.95-kDa PS-Qprot was expressed in *E. coli* cells grown in ^15^N-M9 minimal medium for stable isotope labeling. The tandem hexa-histidine tag at the C-terminus ensured that only fully translated protein species were purified on the Co-NTA column and allowed stringent washes with high imidazole concentrations to efficiently remove impurities. Nevertheless, the PS-Qprot was additionally purified by electrophoresis on an SDS-polyacrylamide gel, followed by electroelution of the protein from the excised gel band. The eluted PS-Qprot was quantified spectroscopically based on the presence of 14 Tyr and 12 Trp residues and correct quantification was verified by separating the PS-Qprot together with a BSA standard on an SDS-polyacrylamide gel and staining with Coomassie blue (**Figure 1B**). The ^15^N-labeled PS-Qprot was first subjected to tryptic digestion alone and peptides were analyzed by LC-MS/MS using a short 6-min gradient to record a total ion count (**Figure 1C**). The latter shows that the Q-peptides separate with characteristic retention times and ion intensities that despite the strict 1:1 stoichiometry of the peptides vary by a factor of about 400. The labeling efficiency, as determined from 20 peptides, was 99.8 ± 0.033% (SD).

**FIGURE 2:**
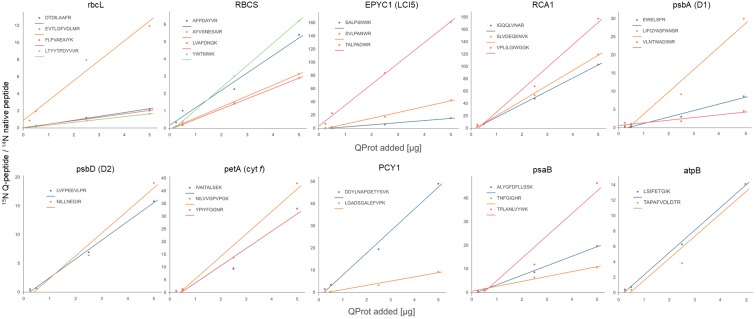
Plots of ratios of ^15^N Q-peptides to ^14^N native peptides versus the amount of PS-Qprot added. Each data point represents the mean of four biological replicates (calculations, values, and SD are compiled in **Supplementary Dataset 1**).

Next, 0.25, 0.5, 2.5, and 5 μg of the ^15^N-labeled PS-Qprot were mixed with 50 μg of (^14^N) whole-cell proteins from mixotrophically grown *Chlamydomonas*. We employed only one preparation of the PS-Qprot, but four independent preparations of *Chlamydomonas* cells. Mixed proteins were precipitated with acetone, followed by tryptic digestion in urea and LC-MS/MS analysis on 45-min analytical gradients. The ion chromatograms of heavy Q-peptide and light native peptide pairs were extracted, surface areas quantified, and ratios calculated (**Supplementary Dataset 1**). The mean ratios ranged from 0.08 for rbcL peptide LTYYTPDYVVR in the sample with the smallest amount of PS-Qprot added to *Chlamydomonas* whole cell proteins up to 178 for RCA1 peptide VPLILGIWGGK in the sample with the largest amount of PS-Qprot added. Nevertheless, plotting the ratios of Q-peptides to native peptides against the amounts of PS-Qprot added revealed generally a linear relationship for each peptide (**Figure 2**). Hence, the range of PS-Qprot added appeared to be well suited for quantifying the photosynthesis target proteins chosen.

### Although Target Protein Peptides Were Quantified Robustly, Their Abundances Varied Considerably Between Different Peptides of the Same Target Protein

Based on the ratios of Q-peptide to native peptide and the known amount of spiked-in PS-Qprot, the abundances of the native peptides in the sample were calculated (in femtomoles per 50 μg of cell proteins) (**Supplementary Dataset 1**). We determined that a *Chlamydomonas* cell of the CC-1883 strain background on average contains 19.9 ± 2.4 pg protein (SD, *n* = 11; **Supplementary Dataset 1**), which allowed us to calculate the absolute amount of each target protein per *Chlamydomonas* cell. Hence, the values shown in **Table 1** represent estimates for the abundance of a target protein in attomol per cell based on 16 individual values for every peptide (four amounts of PS-Qprot added to four independent preparations of whole cell extracts). Although the generally low standard deviations indicate that peptide abundances were estimated robustly, in some cases the obtained abundance values varied considerably between individual peptides of the same protein. This was particularly true for peptides of proteins rbcL, RBCS, EPYC1, psbA, and PCY1. For these peptides, also the slopes of the linear regressions in the plots of the ratios of ^15^N Q-peptide to ^14^N native peptide against PS-Qprot added varied considerably (**Figure 2**). This indicated a consistent over- or underestimation of some native peptides. To aid our interpretation of these results in the Discussion, we used the deep peptide observability predictor (d::pPop) described by Zimmer et al. (unpublished) in this issue to get scores and ranks for estimating the ionization propensities of the Q-peptides employed here (**Table 1**).

**Table 1:** Absolute quantification of major proteins and protein complexes involved in driving the photosynthetic light reactions in the thylakoid membrane and CO_2_ fixation in the pyrenoid.

Protein (complex)		d::pPop rank / score	Peptide	amol/cell^a^	amol/cell^b^	% of total cell protein^c^
rbcL		1 / 1	DTDILAAFR	24 ± 2.7	25.2	6.6
		2 / 0.73	LTYYTPDYVVR	30.7 ± 1.8		
		3 / 0.68	FLFVAEAIYK	22.1 ± 1.6		
		5 / 0.6	(EVTLGFVDLMR	3.2 ± 0.6)^d^		
RBCS1/2		1 / 1	AFPDAYVR	8.1 ± 2.3	16.1	1.3
		2 / 0.996	AYVSNESAIR	17.4 ± 1.5		
		3 / 0.82	LVAFDNQK	21.3 ± 4.1		
		n.d.	YWTMWK	13.8 ± 7.6		
EPYC1 /LCI5		2 / 0.73	SVLPANWR	2.2 ± 1.0	2.0	0.3
		4 / 0.68	SALPSNWK	2.7 ± 1.5		
		7 / 0.53	(TALPADWR	0.3 ± 0.1)		
RCA1		1 / 1	VPLILGIWGGK	0.4 ± 0.1	0.5	0.1
		2 / 0.72	IGQQLVNAR	0.6 ± 0.1		
		11 / 0.32	SLVDEQENVK	0.5 ± 0.1		
PSII	psbA / D1	1 / 1	VLNTWADIINR	8.3 ± 4.5	5.2	2.0^e^
		3 / 0.88	LIFQYASFNNSR	3.1 ± 1.1		
		7 / 0.52	EWELSFR	10.9 ± 5.8		
	psbD / D2	1 / 1	NILLNEGIR	6.3 ± 3.2		
		2 / 0.95	LVFPEEVLPR	4.7 ± 1.7		
		7 / 0.32	(TWFDDADDWLR	n.d.)		
b_6_/*f*	petA / cyt *f*	1 / 1	NILVVGPVPGK	2.3 ± 0.9	2.9	0.5
		2 / 0.97	IVAITALSEK	3.4 ± 1.4		
		6 / 0.54	YPIYFGGNR	3.5 ± 1.5		
PCY1		1 / 1	LGADSGALEFVPK	9.1 ± 2.7	3.5	0.2
		2 / 0.65	DDYLNAPGETYSVK	1.3 ± 0.2		
PSI	psaB	1 / 1	ALYGFDFLLSSK	3.3 ± 0.7	3.1	1.3
		3 / 0.95	TPLANLVYWK	2.5 ± 1.1		
		12 / 0.19	TNFGIGHR	3.6 ± 0.7		
ATP synthase	atpB	3 / 0.94	LSIFETGIK	4.9 ± 1.3	6.1^f^	1.6
		7 / 0.72	TAPAFVDLDTR	8.0 ± 3.4		


*^*a*^Mean ± SD, *n* = 4. ^*b*^Median of all values (*n* = 4 × number of peptides). ^*c*^Based on median and using MWs of mature proteins. ^*d*^Peptides in parentheses were not quantotypic and quantification values therefore omitted. ^*e*^D1 + D2. ^*f*^There are three atpB subunits per ATPase complex, i.e., this number needs to be divided by 3 to obtain the number of ATP synthases per cell.*

To still get a robust estimate for the absolute abundance of the target proteins in the whole cell protein extracts, we used the median of all 8–16 values (each determined with four biological replicates) obtained for the two to four peptides of a target protein (**Table 1**). Furthermore, based on this median value and the molecular weight of the mature protein, the fraction of each target protein in the whole cell protein extract was estimated (**Table 1**). With 25.2 attomol/cell the Rubisco large subunit makes up 6.6% of all proteins in a *Chlamydomonas* cell and with this exceeds the amount of the small subunit by a factor of 1.56. EPYC1 is eight times less abundant than its proposed interaction partner in pyrenoids, RBCS (Meyer et al., 2012; Mackinder et al., 2016) and Rubisco activase is 32-times less abundant than RBCS. Over all, abundances of Rubisco large subunit and Rubisco activase differ by a factor of 50. In contrast, abundances of protein (complexes) involved in photosynthetic light reactions differ far less. Of these, PS II with 5.2 attomol/cell is most abundant, followed by plastocyanin, PS I and the cytochrome b_6_/*f* complex, which range between 2.9 and 3.5 attomol/cell. The least abundant complex is the ATP synthase with 2 attomol/cell.

## Discussion

In this study, we applied the QconCAT strategy to determine the absolute abundance of 10 membrane-intrinsic and soluble proteins that are involved in driving photosynthetic light reactions in the thylakoid membrane and CO_2_ fixation in the pyrenoid. We could robustly quantify the native peptides, as judged from the generally low standard deviations of the quantification values and the good correlation between ratios of ^15^N Q-peptides to ^14^N native peptides versus PS-Qprot added to *Chlamydomonas* whole cell extracts in four dilutions (**Table 1** and **Figure 2**). However, for half of our target proteins we observed a strong variability between the quantification values obtained for different peptides of the same protein, indicating a consistent over- or underestimation of individual native peptides.

### Quantification Problems Arising From Flanking Dibasic Cleavage Sites

The reason for some of these quantification problems are obvious. For example, when the native peptide is flanked at its N-terminus by a dibasic cleavage site and tryptic cleavage was assumed to take place after the second base. Here tryptic cleavage must have occurred exclusively or almost exclusively between the two bases. This explains why we failed to detect psbD peptide K/RTWFDDADDWLR with Q-peptide TWFDDADDWLR and why values obtained for EPYC1 peptide K/RTALPADWR monitored with Q-peptide TALPADWR were at least sixfold lower than expected from the values obtained for the other two EPYC1 peptides employed (**Table 1**; see **Supplementary Dataset 2** for the native context of the target peptides). Nearly quantitative cleavage between two basic residues also explains why quantification values for RCA1 peptide SLVDEQENVK/R and petA peptides IVAITALSEK/K and NILVVGPVPGK/K, all monitored with Q-peptides ending with K, appeared accurate as judged from the values obtained for other peptides from these proteins. Note that d::pPop assigned scores below 0.53 and low ranks to the malperforming peptides TWFDDADDWLR and TALPADWR. The petA peptides – apparently performing well despite being flanked with dibasic cleavage sites – received top scores/ranks, while the apparently also well-performing RCA1 peptide only got a score of 0.32 and rank 11 (**Table 1**).

### Quantification Problems Arising From Methionines and Flanking Acidic Residues

In addition to flanking dibasic cleavage sites, peptides with methionines were recommended to be avoided in QconCAT proteins because methionine can get oxidized to methionine sulfoxide (Brownridge et al., 2011). Two peptides of our QconCAT protein contained methionines. Peptide EVTLGFVDLMR in rbcL gave an abundance value that was at least sevenfold lower than those obtained for the other three peptides chosen for this protein (**Table 1**). We hypothesized that the underestimation of the native peptide was due to it undergoing severe methionine oxidation. However, assuming similar ionization properties for the non-oxidized and oxidized forms of the peptide, we estimated that only 42 ± 22% of the native peptide was oxidized, while this was the case for 67 ± 14% of the Q-peptide (**Supplementary Dataset 1**). As methionines in proteins may get oxidized during sample preparation and SDS gel electrophoresis (Ghesquiere and Gevaert, 2014), our purification of the PS-Qprot by SDS-PAGE and electroelution presumably accounts for the higher extent of methionine oxidation in the rbcL Q-peptide. These data clearly indicate that different extents of methionine oxidation in native and Q-peptides will impair quantification and therefore support the recommendation to avoid methionine-containing peptides in QconCAT proteins (Brownridge et al., 2011; Scott et al., 2016).

The higher oxidation level of the methionine-containing rbcL Q-peptide should lead to an overestimation of the native peptide, but we observe a dramatic underestimation. Moreover, the methionine-containing RBCS peptide gives quantification values close to those obtained for other RBCS peptides. Therefore, the strong underestimation of the EVTLGFVDLMR rbcL peptide must have another reason. In fact, a DD motif follows the arginine in the native context of this peptide (**Supplementary Dataset 2**). Glutamate and aspartate close to tryptic cleavage sites (especially if located at the second position after the cleavage site) have been reported to lead to missed cleavages (Brownridge and Beynon, 2011; Brownridge et al., 2011). We therefore assume that the DD motif present in the context of the native protein, but not in that of the QconCAT protein, was why we underestimated the abundance of the native peptide. Note that out of the four rbcL peptides selected, peptide EVTLGFVDLMR received the lowest score (0.6) from d::pPop (**Table 1**).

Also problematic is PCY1 peptide DDYLNAPGETYSVK. Because it contains the DD motif next to the N-terminal tryptic cleavage site, missed cleavages are likely to take place in the QconCAT protein as well as in the native protein. Assuming an equally incomplete digestion in both, quantification values may still be accurate. However, missed cleavages will also affect the peptide placed N-terminally to DDYLNAPGETYSVK in the QconCAT protein, which is the second PCY1 peptide employed (LGADSGALEFVPK) (**Figure 1**). As this peptide is in a different context in native plastocyanin (**Supplementary Dataset 2**), where it is likely not subject to missed cleavages, we would have overestimated the abundance of the native peptide. This would explain approximately sevenfold difference in quantification values obtained for the two PCY1 peptides (**Table 1**). In line with these observations, d::pPop assigned a score of 1.0 to peptide LGADSGALEFVPK and only of 0.6 to peptide DDYLNAPGETYSVK (**Table 1**).

### Quantification Problems Arising for Unknown Reasons

Some Q-peptides gave quantification results differing from those of other Q-peptides from the same protein with none of the obvious explanations applying, i.e., N-terminal dibasic cleavage sites, the presence of methionines, or acidic residues next to the cleavage site. These were RBCS peptide AFPDAYVR and psbA peptide LIFQYASFNNSR, which by d::pPop were ranked first and third with scores of 1.0 and 0.88, respectively (**Table 1**). We have three possible explanations for this: first, the native peptides might contain posttranslational modifications. Second, they tertiary structures of some proteins might be retained under our digestion conditions and therefore bury tryptic cleavage sites. Third, there might be peptides derived from other proteins in the complex *Chlamydomonas* cell extract that are isobaric with the labeled Q-peptide or the unlabeled native peptide and therefore influence the extracted ion chromatograms used for quantification.

### Comparison of Absolute Quantification Results Obtained for *Chlamydomonas* Proteins

The use of two to four Q-peptides per target protein on the one hand allowed us to pinpoint the described problems with the quantification of individual peptides, because their quantification values could be identified as outliers. On the other hand, despite these outliers, the use of several Q-peptides per target protein allowed a robust quantification of that target protein (**Tables 1, 2**). Only few studies have yet reported absolute protein abundances in *Chlamydomonas*. The by far most comprehensive one reports abundances of 89 proteins and protein complexes based on spiked-in synthetic, isotope-labeled peptides and LC-MS analysis by SRM (Wienkoop et al., 2010). That study covered all proteins and protein complexes analyzed here, except for PS I and EPYC1. While average abundances reported for plastocyanin, rbcL and PS II deviated by less than factor 1.7 from those determined by us with the QconCAT approach, average abundances reported for atpB, the cytochrome b_6_/*f* complex, RBCS and RCA1 were 5–14 times lower (**Table 2**). Another study quantified Rubisco during a diurnal time course with an improved SRM approach (Recuenco-Munoz et al., 2014). The ratios between rbcL and RBCS of 11–44:1 and 5:1 determined in the respective earlier studies compare to a ratio of 1.56:1 determined by us (**Table 2**). The different ratios are mainly attributable to different abundances determined for RBCS, which is surprising because all three studies have used the same RBCS target peptides AYVSNESAIR and LVAFDNQK with d::pPop scores of 0.996 and 0.88, respectively (**Table 1**). Plant Rubisco is composed of eight chloroplast encoded large subunits (rbcL) and eight nucleus-encoded small subunits (RBCS), which are subject to assembly dependent translational regulation, also termed control by epistasy of synthesis (CES) (Wostrikoff and Stern, 2007). Here, rbcL exerts a feedback control on its own synthesis, i.e., unassembled rbcL subunits prevent the translation of additional rbcL subunits if RBCS subunits are limiting (Khrebtukova and Spreitzer, 1996). This implies that rbcL and RBCS are present in a 1:1 stoichiometry, perhaps with a slight excess of rbcL in an unassembled form that serves in repressing its own translation. Although we do not know the truth, in light of the CES process the ratios of rbcL:RBCS of 11–44:1 or 5:1 as reported earlier appear unrealistic and, although much closer to the expected value, even the ratio of 1.56:1 determined by us might be slightly too high.

**Table 2:** Comparison of abundances and ratios of proteins and protein complexes involved in photosynthesis reported for *Chlamydomonas reinhardtii* and pea.

Number of protein (complexes) per *Chlamydomonas* cell									Ratio between complexes/subunits		Reference
		
PSII	PSI	Cyt b_6_/*f*	PCY1	ATP synthase	rbcL	RBCS	RCA1	EPYC1	PSII:PSI:b_6_/*f*	rbcL:RBCS	
1.4–2.5 × 10^6^ (PSBO, P, Q)	–	0.9–1.6 × 10^5^ (PETC)	1–5 × 10^6^	0.2–1.2 × 10^6^ ^b^ (atpB)	2.0–2.6 × 10^7^	0.6–1.7 × 10^6^	4–5 × 10^4^	–	9–29:n:1	11–44:1	Wienkoop et al., 2010^a^
–	–	–	–	–	–	–	–	–	–	5:1	Recuenco-Munoz et al., 2014
–	–	–	–	–	–	–	–	–	1.9:0.6:1 PSI light 2.7:1.4:1 PSII light	–	Murakami et al., 1997
–	–	–	–	–	–	–	–	–	2.3:0.9:1 PSI light 1.9:1.7:1 PSII light^c^	–	Chow et al., 1990
–	–	–	8.2 × 10^6^	–	–	–	–	–	–	–	Merchant et al., 1991
–	–	–	–	–	–	–	–	–	1:1.3:n ^d^	–	Nikolova et al., 2018
3.1 × 10^6^ (psbA, psbD)	1.8 × 10^6^ (psaB)	1.7 × 10^6^ (petA)	2.1 × 10^6^	3.7 × 10^6^ ^b^ (atpB)	1.5 × 10^7^	1.0 × 10^7^	3.0 × 10^5^	1.2 × 10^6^	1.8:1.1:1	1.56:1	This study
**IBAQ rank of indicated protein among 1,207 soluble *Chlamydomonas* proteins**											
3 (PSBO) 13 (PSBQ)	20 (PSAG) 69 (PSAF)	–	1	21 (atpA) 26 (atpB)	2	6	248	153			Schroda et al., 2015


*^*a*^Values vary dependent on mixotrophic or photoautotrophic growth. ^*b*^There are three atpB SU per ATPase complex, i.e., this number needs to be divided by 3 to obtain the number of ATP synthases per cell. ^*c*^Pea thylakoids. ^*d*^Aerobic conditions.*

Also regarding the stoichiometries of the major thylakoid membrane complexes involved in linear electron transport we observe large differences between the results obtained by Wienkoop et al. (2010) and us. Note that quantification was based on different target proteins, i.e., PSBO, PSBP, and PSBQ versus psbA and psbD for PSII, and PETC versus petA for the cytochrome b_6_/*f* complex. While Wienkoop et al. (2010) reported a ratio for PSII:cytochrome b_6_/*f* complex of 9–29:1, we determined a ratio for PSII:PSI:cytochrome b_6_/*f* complex of 1.8:1.1:1. The latter fits well with ratios determined previously by spectroscopy in *Chlamydomonas* cells (Murakami et al., 1997) and in pea thylakoids (Chow et al., 1990) (**Table 2**). Also the ratio for PSI:plastocyanin of 1:1.4, determined in a previous study based on spiked-in purified proteins, heavy labeled cell extracts and mass spectrometry (Nikolova et al., 2018), fits well to the ratio of 1:1.2 determined here by the QconCAT approach (**Table 2**). MS^2^-based quantification via SRM as performed by Wienkoop et al. (2010) and Recuenco-Munoz et al. (2014) is expected to produce more accurate results than the MS^1^-based quantification used here by us because of the high selectivity of MS^2^-based quantification (Lange et al., 2008). So, what might be the reason for the differences observed between the three studies? All studies used very similar digestion protocols, thus ruling out incomplete digestion as a possible cause. A striking difference, however, are different protocols for the extraction of cellular proteins: in the studies by Wienkoop et al. (2010) and Recuenco-Munoz et al. (2014), frozen cell pellets were homogenized in a mortar in the presence of a detergent-free extraction buffer and supernatants obtained after centrifugation were used for absolute quantification. This may have resulted in incomplete extraction of some proteins (RBCS, RCA1, PETC, atpB) while others were well extracted (rbcL, PCY1, PSBO, PSBP, PSBQ). In contrast, we directly added acetone to cell suspensions to directly precipitate all cellular proteins and avoid any prefractionation steps.

### Conclusions and Recommendations for Absolute Quantification via QconCAT Proteins

We conclude that absolute quantification of cellular proteins is no easy task. However, the QconCAT approach appears suitable if care is taken to avoid the many potential pitfalls associated with it. From what we have learned in this study, we recommend the following workflow: first, d::pPop trained on the target organism should be run on the chosen target proteins. Next, the peptides with the highest d::pPop scores should be inspected manually following the rules forwarded previously (Brownridge et al., 2011; Scott et al., 2016) to avoid in this order: (i) peptides harboring acidic residues next to their cleavage sites; (ii) peptides flanked with dibasic residues; (iii) peptides containing methionines. Ideally, at least three peptides should be selected to also have sufficient quantification values if a peptide malperforms because it gets post-translationally modified, is in a region not readily accessible to trypsin, or is isobaric with other peptides in the complex sample.

Placing a tandem hexa-histidine tag at the C-terminus of the QconCAT protein allows purification with stringent washing steps and the presence of several tryptophanes and tyrosines robust spectroscopic quantification. All target proteins addressed by a QconCAT protein should be in a similar abundance range (the ten ones quantified by the QconCAT protein employed here varied by a factor of about 50). For this, rough quantifications by label-free methods like spectral counting (Liu et al., 2004), the empirical abundance index (Ishihama et al., 2005), or intensity-based absolute quantification (IBAQ) (Schwanhausser et al., 2011) give a good guideline. Accordingly, ranks of soluble *Chlamydomonas* proteins rbcL, RBCS, PCY1, EPYC1, and RCA1 determined previously by IBAQ (Schroda et al., 2015) fitted roughly with the abundances determined here with the QconCAT approach (**Table 2**). Finally, no prefractionation steps should be included to avoid an incomplete extraction of some proteins.

## Author Contributions

FS designed the QconCAT protein and performed the LC-MS/MS analyses. AH and DZ analyzed the data. TM and MS conceived and supervised the work. MS wrote the article with contributions from all other authors.

## Conflict of Interest Statement

The authors declare that the research was conducted in the absence of any commercial or financial relationships that could be construed as a potential conflict of interest.

## Footnotes

1 https://web.expasy.org/protparam

2 https://github.com/CSBiology
