# Supplementary material for: Absolute Quantification of Major Photosynthetic Protein Complexes in Chlamydomonas reinhardtii Using Quantification Concatamers (QconCATs)
Source: Front Plant Sci. 2018 Aug 30;9:1265. doi: 10.3389/fpls.2018.01265 (PMC6125352; doi:10.3389/fpls.2018.01265)
Supplement: Supplementary file 2 [file Data_Sheet_2.DOCX]

**Supplemental Dataset 2.** Amino acid sequences of mature target proteins analyzed in this study. Peptides which were included in the PS-Qprot are underlined. Residues known to carry posttranslational modifications are shaded in grey. Molecular weights are given in parentheses.

>sp|P00877|RBL_CHLRE Ribulose bisphosphate carboxylase large chain, mature, Chlamydomonas reinhardtii (52312.61)

PQTETKAGAGFKAGVKDYRLTYYTPDYVVR.DTDILAAFRMTPQLGVPPEECGAAVAAESSTGTWTTVWTDGLTSLDRYKGRCYDIEPVPGEDNQYIAYVAYPIDLFEEGSVTNMFTSIVGNVFGFKALRALRLEDLRIPPAYVKTFVGPPHGIQVERDKLNKYGRGLLGCTIKPKLGLSAKNYGRAVYECLRGGLDFTKDDENVNSQPFMRWRDRFLFVAEAIYKAQAETGEVKGHYLNATAGTCEEMMKRAVCAKELGVPIIMHDYLTGGFTANTSLAIYCRDNGLLLHIHRAMHAVIDRQRNHGIHFRVLAKALRMSGGDHLHSGTVVGKLEGEREVTLGFVDLMRDDYVEKDRSRGIYFTQDWCSMPGVMPVASGGIHVWHMPALVEIFGDDACLQFGGGTLGHPWGNAPGAAANRVALEACTQARNEGRDLAREGGDVIRSACKWSPELAAACEVWKEIKFEFDTIDKL

>sp|P00873|RBS1_CHLRE Ribulose bisphosphate carboxylase small chain 1, mature, Chlamydomonas reinhardtii (16254.82)

MMVWTPVNNKMFETFSYLPPLTDEQIAAQVDYIVANGWIPCLEFAEADKAYVSNESAIRFGSVSCLYYDNRYWTMWKLPMFGCRDPMQVLREIVACTKAFPDAYVR.LVAFDNQKQVQIMGFLVQRPKTARDFQPANKRSV

>sp|P08475|RBS2_CHLRE Ribulose bisphosphate carboxylase small chain 2, mature, Chlamydomonas reinhardtii (16281.81)

MMVWTPVNNKMFETFSYLPPLSDEQIAAQVDYIVANGWIPCLEFAESDKAYVSNESAIRFGSVSCLYYDNRYWTMWKLPMFGCRDPMQVLREIVACTKAFPDAYVR.LVAFDNQKQVQIMGFLVQRPKSARDWQPANKRSV

>sp|P07753|PSBA_CHLRE Photosystem II protein D1, mature, Chlamydomonas reinhardtii (38118.66)

TAILERRENSSLWARFCEWITSTENRLYIGWFGVIMIPCLLTATSVFIIAFIAAPPVDIDGIREPVSGSLLYGNNIITGAVIPTSNAIGLHFYPIWEAASLDEWLYNGGPYQLIVCHFLLGVYCYMGREWELSFRLGMRPWIAVAYSAPVAAASAVFLVYPIGQGSFSDGMPLGISGTFNFMIVFQAEHNILMHPFHMLGVAGVFGGSLFSAMHGSLVTSSLIRETTENESANEGYRFGQEEETYNIVAAHGYFGRLIFQYASFNNSRSLHFFLAAWPVIGIWFTALGLSTMAFNLNGFNFNQSVVDSQGRVLNTWADIINRANLGMEVMHERNAHNFPLDLA

>sp|P06541|ATPB_CHLRE ATP synthase subunit beta, mature, Chlamydomonas reinhardtii (51908.71)

SDSIETKNMGRIVQIIGPVLDIVFAKGQVPNIYNALTIRAKNSAGTEMAVTCEVQQLLGDNCVRAVSMNPTEGLMRGMEVVDTGKPLSVPVGKVTLGRIFNVLGEPVDNMGNVKVEETLPIHRTAPAFVDLDTR.LSIFETGIKVVDLLAPYRRGGKIGLFGGAGVGKTVLIMELINNIAKAHGGVSVFAGVGERTREGNDLYTEMKESGVIVEKNLSDSKVALVYGQMNEPPGARMRVALTALTMAEYFRDVNKQDVLFFIDNIFRFVQAGAEVSALLGRMPSAVGYQPTLATEMGGLQERITSTKDGSITSIQAVYVPADDLTDPAPATTFAHLDATTVLSRNLAAKGIYPAVDPLESTSTMLQPWILGEKHYDSAQSVKKTLQRYKELQDIIAILGLDELSEEDRLIVARARKIERFLSQPFFVAEVFTGSPGKYVSLAETIEGFGKIFAGELDDLPEQAFYLVGNITEAISKAASLK

>tr|A8JH68|A8JH68_CHLRE Plastocyanin, mature, Chlamydomonas reinhardtii (10137.29)

TVKLGADSGALEFVPKTLTIKSGETVNFVNNAGFPHNIVFDEDAIPSGVNADAISRDDYLNAPGETYSVKLTAAGEYGYYCEPHQGAGMVGKIIVQ

>Cre10.g436550.t1.2 LCI5, EPYC1, mature, Chlamydomonas reinhardtii (28053.59)
ASRASSATNRVSPTRSVLPANWRQELESLRNGNGSSSAASSAPAPARSSSASWRDAAPASSAPARSSSASKKAVTPSRSALPSNWKQELESLRSSSPAPASSAPAPARSSSASWRDAAPASSAPARSSSSKKAVTPSRSALPSNWKQELESLRSSSPAPASSAPAPARSSSASWRDAAPASSAPARSSSASKKAVTPSRSALPSNWKQELESLRSNSPAPASSAPAPARSSSASWRDAPASSSSSSADKAGTNPWTGKSKPEIKRTALPADWRKGL

>sp|P23577|CYF_CHLRE Cytochrome f, mature, Chlamydomonas reinhardtii (31249.09)

YPVFAQQNYANPREANGRIVCANCHLAQKAVEIEVPQAVLPDTVFEAVIELPYDKQVKQVLANGKKGDLNVGMVLILPEGFELAPPDRVPAEIKEKVGNLYYQPYSPEQKNILVVGPVPGKKYSEMVVPILSPDPAKNKNVSYLKYPIYFGGNRGRGQVYPDGKKSNNTIYNASAAGKIVAITALSEKKGGFEVSIEKANGEVVVDKIPAGPDLIVKEGQTVQADQPLTNNPNVGGFGQAETEIVLQNPARIQGLLVFFSFVLLTQVLLVLKKKQFEKVQLAEMNF

>sp|P09144|PSAB_CHLRE Photosystem I P700 chlorophyll a apoprotein A2, mature, Chlamydomonas reinhardtii (82108.75)

MATKLFPKFSQGLAQDPTTRRIWYGLAMAHDFESHDGMTEENLYQKIFASHFGQLSIIFLWTSGNLFHVAWQGNFEQWVTDPVHIRPIAHAIWDPHFGQPAVEAFTRGGASGPVNISTSGVYQWWYTIGMRTNQDLYVGSVFLALVSAIFLFAGWLHLQPNFQPSLSWFKDAESRLNHHLSGLFGVSSLAWTGHLVHVAIPESRGQHVGWDNFLSVLPHPQGLTPFFTGNWAAYAQSPDTASHVFGTAQGSGQAILTFLGGFHPQTQSLWLTDMAHHHLAIAVIFIVAGHMYRTNFGIGHRMQAILEAHTPPSGSLGAGHKGLFDTVNNSLHFQLGLALASVGTITSLVAQHMYSLPPYAFQAIDFTTQAALYTHHQYIAGFIMCGAFAHGAIFFIRDYDPEQNKGNVLARMLDHKEALISHLSWVSLFLGFHTLGLYVHNDVMQAFGTPEKQILIEPVFAQWIQAAHGKALYGFDFLLSSKTSAAFANGQSLWLPGWLDAINNNQNSLFLTIGPGDFLVHHAIALGLHTTTLILVKGALDARGSKLMPDKKDFGYSFPCDGPGRGGTCDISAYDAFYLAVFWMLNTIGWVTFYWHWKHLTLWQGNVAQFDESSTYLMGWLRDYLWLNSSQLINGYNPFGMNSLSVWAWTFLFGHLIYATGFMFLISWRGYWQELIETLVWAHEKTPLANLVYWKDKPVALSIVQARLVGLAHFSVGYIFTYAAFLIASTSGRFG

>sp|P06007|PSBD_CHLRE Photosystem II D2 protein, mature, Chlamydomonas reinhardtii (39316.11)

TIAIGTYQEKRTWFDDADDWLRQDRFVFVGWSGLLLFPCAYFALGGWLTGTTFVTSWYTHGLATSYLEGCNFLTAAVSTPANSMAHSLLFVWGPEAQGDFTRWCQLGGLWAFVALHGAFGLIGFMLRQFEIARSVNLRPYNAIAFSAPIAVFVSVFLIYPLGQSGWFFAPSFGVAAIFRFILFFQGFHNWTLNPFHMMGVAGVLGAALLCAIHGATVENTLFEDGDGANTFRAFNPTQAEETYSMVTANRFWSQIFGVAFSNKRWLHFFMLLVPVTGLWMSAIGVVGLALNLRAYDFVSQEIRAAEDPEFETFYTKNILLNEGIRAWMAAQDQPHERLVFPEEVLPRGNAL

>Cre04.g229300.t1.1, RCA1, mature, Chlamydomonas reinhardtii (41532.15)

ASSRKQMGRWRSIDAGVDASDDQQDITRGREMVDDLFQGGFGAGGTHNAVLSSQEYLSQSRASFNNIEDGFYISPAFLDKMTIHIAKNFMDLPKIKVPLILGIWGGKGQGKTFQCALAYKKLGIAPIVMSAGELESGNAGEPAKLIRTRYREASDIIKKGRMCSLFINDLDAGAGRMGDTTQYTVNNQMVNATLMNIADNPTNVQLPGVYKNEEIPRVPIVCTGNDFSTLYAPLIRDGRMEKYYWNPTREDRIGVCMGIFQEDNVQRREVENLVDTFPGQSIDFFGALRARVYDDMVRQWITDTGVDKIGQQLVNARQKVAMPKVSMDLNVLIKYGKSLVDEQENVKRVQLADAYLSGAELAGHGGSSLPEAYSR
